# Relationship Between Preoperative Neutrophil-to-Lymphocyte Ratio and Patient-Controlled Analgesia Consumption Following Cardiac Surgery: A Retrospective Study

**DOI:** 10.7759/cureus.88230

**Published:** 2025-07-18

**Authors:** Takuya Akiyama, Yosuke Nakadate, Emi Nakajima, Tetsuya Iijima, Takashi Matsukawa

**Affiliations:** 1 Anesthesiology, University of Yamanashi, Kofu, JPN; 2 Anesthesiology, University of Tsukuba, Tsukuba, JPN

**Keywords:** cardiac surgery, cardiopulmonary bypass, intravenous patient-controlled analgesia, neutrophil-to-lymphocyte ratio, postoperative analgesic consumption, preoperative inflammatory index

## Abstract

Background

The neutrophil-to-lymphocyte ratio (NLR), an inflammatory marker, is known to predict postoperative complications. However, its predictive value for postoperative inflammatory status and postoperative pain (POP) following cardiac surgery remains unclear. This study aimed to determine whether elevated preoperative NLR is associated with increased postoperative analgesic consumption.

Methods

We retrospectively analyzed patients aged over 18 years who underwent cardiac surgery and received intravenous patient-controlled analgesia (IVPCA) for POP between April 2017 and March 2019. The IVPCA solution contained fentanyl (5 μg/mL), lidocaine (8 mg/mL), ketamine (0.8 mg/mL), and droperidol (50 μg/mL). The primary outcome was the correlation between preoperative NLR and postoperative IVPCA consumption. Patients were categorized into high (> median) and low (< median) preoperative NLR groups. To adjust for baseline differences, propensity score matching was applied. Post-matching, we compared perioperative variables and reassessed the association between preoperative NLR and IVPCA usage.

Results

A total of 131 patients were included. The median preoperative NLR was 2.29 (IQR: 1.58-3.39). Across all patients, preoperative NLR was not correlated with postoperative IVPCA consumption (ρ = -0.08, P = 0.34). After excluding the patient with the median NLR, 76 patients (38 in each group) remained for analysis post-matching. IVPCA consumption was comparable between the high and low NLR groups (108.6 (77.6-164.0) mL vs. 97.1 (83.1-148.0) mL, P = 0.97). However, patients with higher preoperative NLR had significantly elevated NLR on postoperative days 2 and 3 compared to those with lower preoperative NLR (11.0 (7.8-16.4) vs. 7.4 (6.2-10.8), P = 0.02; and 11.3 (7.5-17.3) vs. 6.6 (3.3-4.6), P = 0.008, respectively). Despite this, preoperative NLR was not associated with any clinical outcomes.

Conclusions

Preoperative NLR was not predictive of IVPCA consumption or clinical outcomes following cardiac surgery.

## Introduction

Despite ongoing advancements in surgical techniques, anesthetic agents, and perioperative monitoring, morbidity and mortality rates in cardiac surgery patients remain higher than in those undergoing other surgical procedures [1]. Postoperative pain (POP), which results from acute inflammatory responses to surgical trauma, including sternal fracture and dislocation, artery dissection, tissue retraction, and vein harvesting [2], is not only a distressing symptom but also a potential contributor to postoperative complications. It can impair respiratory function, hinder effective coughing, increase the risk of pulmonary infections, and lead to hemodynamic instability and cardiac overload, such as elevated oxygen demand and a heightened risk of myocardial ischemia [3]. Inadequate pain control may prolong ICU stays, increase in-hospital morbidity and mortality, and contribute to the development of chronic pain [4,5]. Therefore, effective POP management is critical to improving postoperative outcomes in cardiac surgery patients [4,5].

Intravenous patient-controlled analgesia (IVPCA) is one approach to managing POP [6]. Delivered through an intravenous catheter and pump, IVPCA allows the clinician to set parameters such as the basal infusion rate, bolus dose, and lockout interval. Compared to conventional continuous infusion, IVPCA may provide more effective analgesia by allowing patients a degree of control over their pain relief. Additionally, analgesic consumption via IVPCA serves as an indirect yet objective measure of pain intensity [7]. Nevertheless, the optimal IVPCA settings for cardiac surgery patients remain undetermined.

The neutrophil-to-lymphocyte ratio (NLR), a widely recognized marker of systemic inflammation [8], has garnered growing interest. Preoperative inflammatory markers like NLR have been used to predict postoperative inflammatory responses in orthopedic procedures such as hip and knee surgeries [9]. Furthermore, elevated systemic inflammation has been linked to acute POP following sternotomy [10]. Evaluating whether NLR can predict postoperative IVPCA consumption in cardiac surgery patients may help guide individualized IVPCA settings and improve pain management in this population.

In this study, we tested the hypothesis that a higher preoperative NLR would be associated with increased postoperative analgesic consumption in patients undergoing cardiac surgery. We also explored the potential of preoperative NLR as a tool to personalize IVPCA parameters for more effective POP management.

## Materials and methods

Ethics statement

This retrospective study was approved by the University of Yamanashi Ethics Committee (approval number: 2108; approval date: 27 August 2019). The study adhered to the Strengthening the Reporting of Observational Studies in Epidemiology (STROBE) guidelines. Given its retrospective nature, the requirement for informed consent was waived.

Patients

Patients aged 18 years or older who underwent scheduled cardiac surgery requiring cardiopulmonary bypass (CPB) between April 1, 2017, and March 31, 2019, and received IVPCA for postoperative analgesia were eligible for inclusion. Exclusion criteria were emergency operations, major vascular surgeries, and missing data. Emergency procedures were excluded due to the patients’ unstable condition, which could alter the preoperative inflammatory status compared to elective cases. Additionally, major vascular surgeries were excluded because they often require hypothermia, resulting in significantly different perioperative characteristics from those of valve surgery or coronary artery bypass grafting. Of the 240 patients who underwent cardiovascular surgery during the study period, 154 met the inclusion criteria.

Anesthesia protocol

General anesthesia was induced using midazolam, fentanyl, and rocuronium. Maintenance was achieved with sevoflurane or propofol, a continuous infusion of remifentanil, and intermittent administration of fentanyl and rocuronium. In addition to standard monitoring, patients underwent cerebral regional oxygen saturation monitoring, invasive arterial pressure monitoring (radial), pulmonary artery catheterization, central venous pressure measurement, and transesophageal echocardiography. Prior to CPB initiation, 300 IU/kg of heparin was administered, with activated clotting time (ACT) maintained above 480 seconds. During CPB, body temperature was maintained between 32 and 34°C, and mean arterial pressure between 50 and 80 mmHg. After CPB, anticoagulation was reversed using protamine to achieve ACT <120 seconds.

Postoperatively, patients were kept intubated, sedated, and mechanically ventilated in the ICU. Sedation was maintained with either propofol or dexmedetomidine infusions. Extubation was performed once patients became normothermic, hemodynamically stable, conscious, and capable of spontaneous breathing. Further postoperative care was at the discretion of the attending surgeons.

IVPCA and postoperative patient management

A multimodal IVPCA regimen was used, consisting of fentanyl (5 μg/mL), lidocaine (8 mg/mL, preservative-free), ketamine (0.8 mg/mL), and droperidol (50 μg/mL), following previously reported protocols [11,12]. The IVPCA device (i-Fuser plus™, JMS Co., Ltd., Tokyo, Japan) was programmed to deliver a basal rate of 4 mL/h and a 3-mL bolus with a 10-minute lockout interval. IVPCA infusion was initiated during CPB. Postoperative IVPCA settings and any additional analgesics (e.g., acetaminophen or NSAIDs) were adjusted at the surgeon’s discretion.

Data collection

Data were obtained from patients’ medical and anesthesia records, as well as IVPCA documentation. Demographic data included age, sex, height, weight, and comorbidities, along with preoperative laboratory values. Surgical details such as procedure type, operation time, anesthesia time, and CPB duration were recorded. Postoperative data included duration of intubation, length of ICU and hospital stay, 30-day mortality, and major morbidities [13], defined as any of the following: cardiac reoperation, deep sternal wound infection or mediastinitis, permanent stroke, prolonged mechanical ventilation, or renal failure.

IVPCA usage data included total drug volume consumed, usage duration, number of bolus attempts (first 24 hours and total), and number of successful bolus deliveries (first 24 hours and total). Data with missing values were excluded to ensure accuracy.

Outcomes

The primary outcome was the correlation between preoperative NLR and total postoperative IVPCA consumption. Secondary outcomes included correlations between preoperative NLR and other IVPCA parameters, duration of postoperative intubation, ICU stay, hospital stay, and postoperative complications.

Patients were divided into high and low NLR groups based on the median preoperative NLR value, as no standard cut-off has been universally accepted [14]. Perioperative variables were compared between these two groups. To minimize confounding, propensity score matching was performed based on preoperative variables.

Statistical analysis

Continuous variables are presented as mean ± SD or median (IQR), and categorical variables as counts (percentages). Relationships between preoperative NLR and postoperative variables were assessed using Spearman’s rank correlation coefficient. Group comparisons were performed using the Mann-Whitney U test or Student’s t test for continuous variables and the chi-square or Fisher’s exact test for categorical variables.

To control for differences in baseline characteristics, propensity score matching was conducted using logistic regression, including all preoperative variables with a P value <0.1. A nearest-neighbor matching algorithm with a 1:1 ratio and a caliper width of 0.2 for the logit-transformed score was applied. A P value <0.05 was considered statistically significant. Subgroup analyses were conducted if significant differences in comorbidities were observed between the two NLR groups.

A sample size of at least 118 patients was required to achieve 90% statistical power at a 5% significance level to detect a Spearman’s correlation coefficient of 0.3 between preoperative NLR and postoperative IVPCA consumption.

All analyses were performed using IBM SPSS Statistics for Windows, Version 29.0 (Released 2022; IBM Corp., Armonk, NY, USA).

## Results

**Figure 1:**
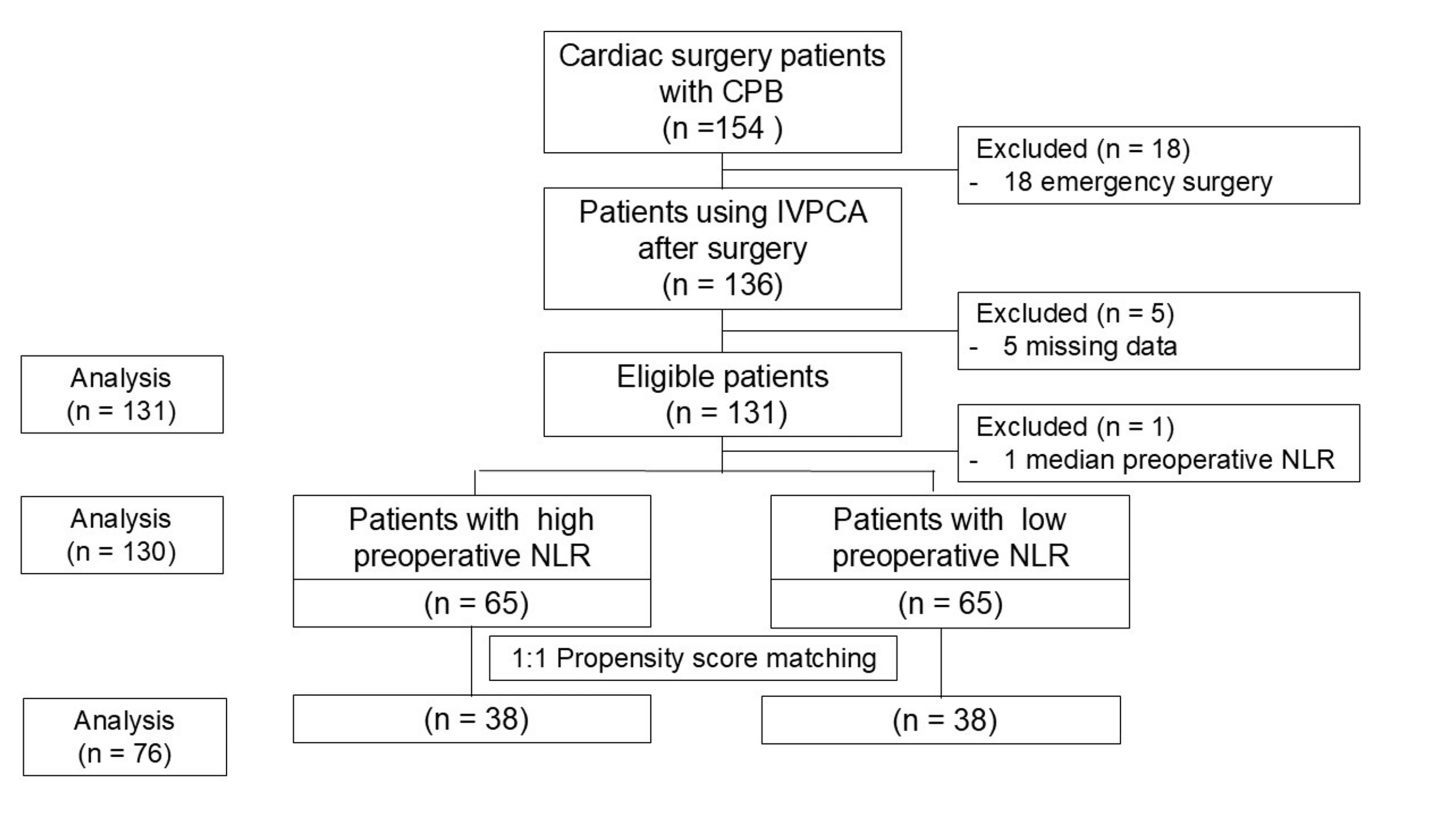
Flow diagram of patient inclusion in the study Of the 131 eligible patients, one patient with the median preoperative NLR value (2.29) was excluded from further analysis to ensure equal group sizes. CPB: cardiopulmonary bypass; IVPCA: intravenous patient-controlled analgesia; NLR: neutrophil-to-lymphocyte ratio

A total of 154 patients underwent cardiac surgery with CPB during the study period. After excluding 18 patients who underwent emergency procedures and five patients with missing data, 131 patients were included in the final analysis (Figure 1).

The mean age of the 131 patients was 71.1 years, and 40 (30.5%) were female (Table 1). All patients underwent cardiac surgery via total median sternotomy. The types of surgical procedures are summarized in Table 1. The median preoperative NLR was 2.29 (IQR: 1.58-3.39). Patients received IVPCA with a total volume of 105.5 mL (80.7-152.4), corresponding to a fentanyl consumption of 527.5 µg (403.5-762.0), administered over a median duration of 42.5 hours (38.5-53.8). The number of bolus requests was 3.0 (1.0-8.0) during the first 24 hours and 10.0 (3.0-18.0) in total, while the number of bolus deliveries was 3.0 (1.0-7.0) in the first 24 hours and 9.0 (3.0-17.0) overall (Table 2). No significant correlation was found between preoperative NLR and postoperative IVPCA consumption (ρ = -0.08, P = 0.34), nor with any other IVPCA usage parameters (Table 3).

**Table 1:** Patient characteristics Data are presented as mean ± SD, median (IQR), or counts (percentages). Continuous variables were compared using the Mann-Whitney U test or Student’s t test, while categorical variables were analyzed using the chi-squared test or Fisher’s exact test. Test statistics are reported as t, z, or chi-squared values, as appropriate. Alb: albumin; ASA-PS: American Society of Anesthesiologists Physical Status classification; BA: bronchial asthma; BSA: body surface area; Cre: creatinine; DM: diabetes mellitus; Hb: hemoglobin; HbA1c: hemoglobin A1c; Hct: hematocrit; HT: hypertension; LVEF: left ventricular ejection fraction; NLR: neutrophil-to-lymphocyte ratio

Variable	All patients (n = 131)	Total set (n = 65 in each)				Matched set (n = 38 in each)			
		High preoperative NLR (>2.29)	Low Preoperative NLR (<2.29)	Test statistics	P value	High preoperative NLR (>2.29)	Low preoperative NLR	Test statistics	P value
Demographics									
Age (year)	71.1 ± 11.0	73.6 ± 8.7	68.5 ± 12.5	-2.69	0.008	73.1 ± 8.5	72.4 ± 9.5	-0.34	0.73
Male/female (n)	90/41	47/18	43/22	0.58	0.45	25/13	24/14	0.06	0.81
Height (cm)	160.4 ± 9.7	160.7 ± 9.8	160.4 ± 9.6	-0.22	0.83	159.1 ± 10.9	159.9 ± 9.4	0.33	0.74
Weight (kg)	60.1 ± 12.9	58.2 ± 12.0	62.4 ± 13.2	1.88	0.06	58.6 ± 12.9	59.2 ± 11.4	0.22	0.82
BMI (kg/m²)	23.2 ± 3.6	22.4 ± 3.4	24.1 ± 3.7	2.75	0.007	23.0 ± 3.6	23.0 ± 2.8	0.06	0.96
BSA (m²)	1.63 ± 0.21	1.66 ± 0.21	1.61 ± 0.20	1.49	0.14	1.60 ± 0.22	1.62 ± 0.20	0.31	0.76
Comorbidities									
HT (n)	70 (52.2)	34 (52.3)	36 (56.3)	0.2	0.65	19 (50.0)	24 (64.9)	1.69	0.19
DM (n)	48 (36.6)	31 (47.7)	17 (26.2)	6.47	0.01	12 (31.6)	12 (31.6)	0	1
Smoker (n)	62 (48.1)	31 (48.4)	31 (48.4)	0	1	18 (47.4)	17 (45.9)	0.02	0.9
BA (n)	6 (4.6)	2 (3.1)	4 (6.2)	0.7	0.4	0 (0)	2 (5.3)	2.03	0.49
ASA-PS	4 (4-4)	4 (4-4)	4 (4-4)	-0.63	0.53	4 (4-4)	4 (4-4)	-0.84	0.4
LVEF (%)	65.0 (52.7-71.1)	62.5 (45.5-70.7)	67.0 (58.0-72.0)	-1.83	0.07	65.5 (45.0-72.5)	64.0 (53.2-72.7)	-0.46	0.64
Preoperative blood tests									
WBC /×10³/μL)	5.7 (4.8-6.6)	5.8 (4.8-6.9)	5.5 (4.7-6.5)	1.27	0.2	5.8 (5.1-6.9)	5.4 (4.7-6.5)	1.34	0.18
Neutrophil (×10³/μL)	3.5 (2.7-4.4)	4.1 (3.3-4.7)	2.9 (2.2-3.6)	5.55	<0.001	4.3 (3.3-4.7)	2.9 (2.2-3.3)	4.61	<0.001
Lymphocyte (×10³/μL)	1.4 (1.1-2.0)	1.2 (0.9-1.4)	1.9 (1.5-2.4)	-7.22	<0.001	1.2 (0.9-1.4)	1.9 (1.4-2.4)	-5.24	<0.001
Hct (%)	37.8 (34.4-41.4)	37.0 (33.9-40.8)	39.1(36.3-42.3)	-2.42	0.02	37.9 (35.5-41.9)	38.4 (35.7-41.6)	0.14	0.89
Alb (mg/dL)	4.1 (3.7-4.4)	4.0 (3.6-4.3)	4.1 (3.8-4.4)	-2.11	0.04	4.1 (3.8-4.5)	4.1 (3.7-4.3)	0.98	0.33
Cre (mg/dL)	0.9 (0.8-1.3)	1.1 (0.9-1.7)	0.8 (0.7-1.1)	4.55	<0.001	0.9 (0.9-1.2)	0.9 (0.8-1.2)	0.83	0.41
CRP (mg/dL)	0.11 (0.00-0.30)	0.15 (0.00-0.54)	0.00 (0.00-0.25)	2.1	0.04	0.16 (0.00-0.30)	0.00 (0.00-0.26)	0.53	0.6
HbA1c (mg/dL)	5.9 (5.6-6.4)	6.0 (5.5-6.4)	5.8 (5.7-6.2)	0.34	0.73	5.9 (5.6-6.3)	5.9 (5.7-6.4)	-0.61	0.54

**Table 2:** Anesthetic/surgical data and outcomes AVR: aortic valve replacement; CABG: coronary artery bypass graft; CPB: cardiopulmonary bypass; Delta NLR: each day of NLR - preoperative NLR; IVPCA: intravenous patient-controlled analgesia; MVRR: mitral valve repair or replacement; NLR: neutrophil-to-lymphocyte ratio; POD: postoperative day; TAP: tricuspid annuloplasty

Variable	All patients (n = 131)	Total set (n = 65 in each)				Matched set (n = 38 in each)			
		High preoperative NLR (>2.29)	Low preoperative NLR (<2.29)	Test statistics	P value	High preoperative NLR (>2.29)	Low Preoperative NLR (<2.29)	Test statistics	P value
Anesthetic duration (minutes)	395 (344-456)	398 (352-487)	379 (329-445)	1.46	0.14	434 (351-517)	381 (330-447)	1	0.32
Surgical duration (minutes)	331 (270-391)	335 (281-412)	307 (263-383)	1.31	0.19	342 (268-426)	305 (263-380)	0.78	0.43
CPB time (minutes)	157 (127-201)	165 (129-201)	148 (117-202)	1.11	0.27	170 (133-208)	139 (125-204)	1.22	0.22
Surgery type				12.11	0.15			4.55	0.8
Valve									
AVR (n)	40 (30.5)	20 (30.8)	20 (30.8)			12 (31.6)	11 (28.9)		
MVRR (n)	16 (12.2)	3 (4.6)	13 (20.0)			3 (7.9)	5 (13.2)		
TAP (n)	1 (0.8)	0 (0.0)	1 (1.6)			0 (0.0)	1 (2.6)		
Multiple valve (n)	23 (17.6)	15 (23.1)	7 (10.8)			7 (18.4)	4 (10.5)		
CABG	26 (19.8)	15 (23.1)	11 (16.9)			7 (18.4)	6 (15.8)		
CABG + valve									
CABG + AVR (n)	12 (9.1)	6 (9.2)	6 (9.2)			4 (10.5)	5 (13.2)		
CABG + MVRR (n)	1 (0.8)	0 (0.0)	1 (1.6)			0 (0.0)	1 (2.6)		
CABG + multi valve (n)	5 (3.8)	2 (3.1)	3 (4.6)			1 (2.6)	3 (7.9)		
Others	7 (5.4)	4 (6.2)	3 (4.6)			3 (7.9)	3 (7.9)		
Intraoperative opioid administration									
Remifentanil (mcg/kg/min)	0.43 (0.36-0.53)	0.44 (0.36-0.52)	0.43 (0.36-0.54)	-0.21	0.84	0.42 (0.34-0.51)	0.44 (0.34-0.55)	-0.65	0.52
Fentanyl (mcg/kg/hr)	4.1 (3.0-5.6)	3.9 (2.7-5.5)	4.3 (3.2-5.7)	-0.79	0.43	3.9 (2.4-6.1)	4.3 (3.1-5.5)	-0.57	0.59
Postoperative IVPCA usage									
Drug requirement in total (mL)	105.5 (80.7-152.4)	104.5 (77.9-147.6)	106.5 (83.3-164.7)	-1.14	0.25	108.6 (77.6-164.0)	97.1 (83.1-148.0)	0.04	0.97
Usage duration (hours)	42.5 (38.5-53.8)	43.4 (38.9-63.6)	42.4 (38.1-46.3)	1.47	0.14	42.3 (38.5-62.4)	42.3 (38.2-45.3)	0.55	0.58
Number of bolus requests for the first 24 hours	3.0 (1.0-8.0)	2.0 (1.0-6.5)	4.0 (2.0-8.0)	-1.54	0.12	3.5 (0.8-8.0)	4.0 (2.0-6.0)	-0.39	0.7
Number of bolus requests in total	10.0 (3.0-18.0)	9.0 (2.0-20.5)	11.0 (6.0-18.0)	-1.45	0.15	7.0 (2.0-22.0)	9.0 (6.0-17.3)	-0.88	0.38
Number of bolus deliveries for the first 24 hours	3.0 (1.0-7.0)	2.0 (1.0-6.0)	4.0 (2.0-8.0)	-1.62	0.11	3.0 (0.8-8.0)	3.5 (2.0-6.0)	-0.42	0.67
Number of bolus deliveries in total	9.0 (3.0-17.0)	9.0 (2.0-18.0)	10.0 (6.0-17.0)	-1.62	0.11	7.0 (2.0-18.8)	9.0 (6.0-16.3)	-1.11	0.27
Outcomes									
Intubation duration (hours)	15.1 (12.1-17.6)	15.5 (12.5-17.7)	13.3 (10.9-16.9)	1.15	0.25	13.4 (8.8-16.6)	14.6 (9.1-19.0)	-0.81	0.42
Length of ICU stay (day)	3.0 (2.0-3.0)	3.0 (2.0-4.0)	3.0 (2.0-3.0)	0.94	0.35	2.5 (2.0-3.0)	3 (2.0-3.3)	-1.39	0.17
Length of hospital stay	16.0 (13.0-20.0)	17.0 (15.0-20.5)	15.0 (13.0-18.5)	2.51	0.01	17.0 (14.8-21.3)	15.0 (13.0-21.0)	0.92	0.06
Morbidity (n)	19 (14.2)	11 (16.9)	8 (12.5)	0.5	0.48	6 (15.8)	7 (18.9)	0.13	0.72
Reoperation (n)	2 (1.5)	1 (1.5)	1 (1.5)	0	1	1 (2.6)	1 (2.6)	0	1
Stroke (n)	9 (6.9)	7 (10.8)	2 (3.1)	2.96	0.16	4 (10.5)	2 (5.3)	0.71	0.67
Renal failure (n)	1 (0.8)	1 (1.5)	0 (0.0)	1	1	0 (0.0)	0 (0.0)	-	-
Prolonged intubation (n)	14 (10.7)	8 (12.3)	6 (9.2)	0.32	0.57	5 (13.2)	6 (15.8)	0.11	0.74
Surgical site infection (n)	1 (0.8)	0 (0.0)	1 (1.5)	1	1	0 (0.0)	0 (0.0)	-	-
Death (n)	5 (3.7)	3 (4.6)	2 (3.1)	0.21	0.65	2 (5.3)	2 (5.3)	0	1
Delta NLR									
POD0	7.7 (4.1-13.7)	12.6 (5.2-17.0)	5.6 (3.1-11.0)	2.57	0.01	12.6 (5.4-14.7)	4.5 (3.1-13.7)	1.76	0.08
POD1	10.6 (6.7-15.7)	12.4 (7.6-17.0)	9.7 (6.3-13.5)	1.44	0.15	12.5 (8.0-15.4)	9.8 (5.8-16.4)	1.02	0.31
POD2	9.1 (6.7-13.6)	11.1 (7.4-17.2)	8.2 (6.3-10.1)	2.63	0.01	11.0 (7.8-16.4)	7.4 (6.2-10.8)	2.36	0.02
POD3	7.1 (4.9-10.8)	7.8 (5.5-13.8)	5.7 (4.0-9.0)	2.44	0.02	11.3 (7.5-17.3)	6.6 (3.3-9.4)	2.66	0.01
POD5	2.8 (1.7-4.8)	3.7 (2.0-6.5)	2.1 (1.5-3.3)	2.78	0.01	3.2 (1.9-4.4)	2.2 (1.5-4.6)	0.87	0.39
POD7	2.1 (1.4-4.4)	3.1 (1.4-5.8)	2.0 (1.4-3.6)	1.17	0.24	3.6 (1.3-4.9)	2.6 (1.5-4.5)	0.13	0.9

**Table 3:** Correlation between preoperative NLR and IVPCA usage/outcomes The relationship between preoperative NLR and postoperative parameters was analyzed using Spearman's rank correlation coefficient. Delta NLR: each day of NLR - preoperative NLR; IVPCA: intravenous patient-controlled analgesia; NLR: neutrophil-to-lymphocyte ratio; POD: postoperative day

Parameter	All patients (n = 131)		Matched set (n = 76)	
	ρ	P value	ρ	P value
IVPCA usage				
Drug requirement in total (mL)	-0.02	0.91	0.24	0.26
Usage duration (hours)	0.33	0.02	0.41	0.05
Number of bolus requests for the first 24 hours	-0.23	0.12	-0.04	0.85
Number of bolus requests in total	-0.14	0.35	0.05	0.82
Number of bolus deliveries for the first 24 hours	-0.24	0.1	-0.05	0.82
Number of bolus deliveries in total	-0.15	0.3	0.01	0.96
Outcomes				
Intubation duration (hours)	0.25	0.09	0.11	0.6
Length of ICU stay (day)	0.2	0.18	-0.14	0.53
Length of hospital stay (day)	0.13	0.36	0.13	0.56
Delta NLR				
POD 0	0.36	0.11	0.26	0.45
POD 1	0.01	0.98	0.31	0.24
POD 2	0.17	0.38	0.32	0.23
POD 3	0.26	0.27	0.67	0.07
POD 5	0.3	0.13	0.02	0.96
POD 7	-0.07	0.74	-0.14	0.64

One patient with the median preoperative NLR value (2.29) was excluded from further analysis to ensure equal group sizes. Patient characteristics were then compared between those with high (>2.29) and low (<2.29) preoperative NLR, with 65 patients in each group (Table 1). Patients in the high NLR group were older, had lower BMI, and showed higher rates of diabetes mellitus, hypoalbuminemia, renal dysfunction, and anemia (Table 1). To adjust for these differences, age, height, weight, hematocrit, serum creatinine, left ventricular ejection fraction, and a history of diabetes were included in the logistic regression model for propensity score matching. After matching, the baseline characteristics were balanced between groups (Table 1).

All postoperative IVPCA usage parameters, including total consumption, were comparable between the two groups. Postoperative NLR was elevated in both the full cohort and the matched set. Although patients with high preoperative NLR had a longer hospital stay in the full cohort, this difference was not statistically significant after matching (Table 2). Postoperative NLR remained significantly higher in patients with high preoperative NLR compared to those with low NLR. However, preoperative NLR showed no significant correlation with any IVPCA usage parameters or postoperative outcomes (Table 3).

Results for the subgroup of 48 patients with diabetes mellitus are presented in Appendix A, Appendix B, and Appendix C. In this subgroup, preoperative NLR was not correlated with postoperative IVPCA parameters or outcomes, except for IVPCA usage duration, which showed a moderate negative correlation (ρ = -0.41, P = 0.04; Appendix C).

## Discussion

In this study, preoperative NLR did not predict increased postoperative IVPCA consumption or clinical outcomes, although it was associated with elevated postoperative NLR levels. In orthopedic surgery, a higher preoperative NLR has been linked to greater postoperative NLR elevation and worsened POP [9,15,16]. Additionally, preoperative NLR has been shown to predict postoperative analgesic requirements in orthognathic surgery [7]. A heightened preoperative inflammatory state has also been associated with increased postoperative NLR levels in orthopedic procedures [9].

Consistent with these findings, our study observed elevated postoperative NLR in cardiac surgery patients with high preoperative NLR. However, this did not translate to increased IVPCA demand, in contrast to what has been reported in orthopedic surgeries. This discrepancy may stem from differences in POP mechanisms and the sources of intraoperative inflammation between cardiac and orthopedic procedures.

In orthopedic and orthognathic surgeries, POP is primarily due to nociceptive input from injuries to the skin, subcutaneous tissue, cartilage, and bone, with inflammatory mediators released at the surgical site. Primary afferent Aδ fibers transmit these impulses to the dorsal horn of the spinal cord, which then relays sensory information to supraspinal structures where the perception of pain occurs. In contrast, POP in cardiac surgery, particularly following sternotomy, is mainly mediated by the intercostal nerves and pericardial pain fibers. Additionally, prolonged sternal retraction and the use of chest and mediastinal tubes can cause injury to distant anatomical regions. Both C and Aδ fibers from multiple regions contribute to the pain experience [10]. As a result, the mechanisms of POP in cardiac surgery are more complex, involving multiple anatomical areas and neural pathways.

The sources of postoperative inflammation also differ. In orthopedic procedures, the inflammatory response is primarily localized to the surgical site [17]. Cardiac surgery, on the other hand, involves sternotomy, direct cardiac incision, and CPB, a procedure unique to cardiac operations that exposes blood to the foreign surfaces of an extracorporeal circuit, triggering systemic inflammatory response syndrome (SIRS) [18]. CPB-induced inflammation involves numerous immune cascades and cellular responses, including neutrophil activation. Although postoperative NLR levels in orthopedic patients typically range from 8 to 10 [19,20], similar to those observed in this study, the mechanisms driving these levels are fundamentally different.

**Figure 2:**
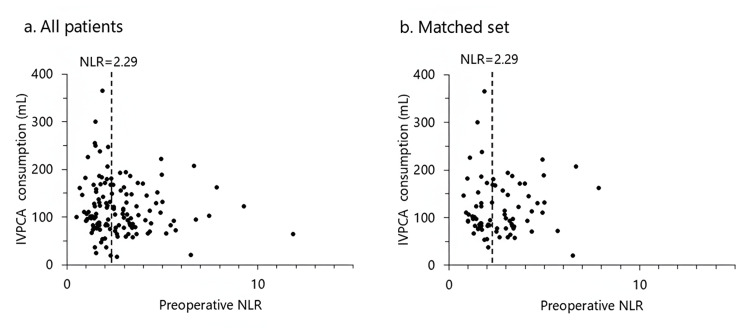
Relationship between preoperative NLR and postoperative IVPCA consumption (a) Scatter plot for all patients (ρ = -0.08, P = 0.34; two-sided Spearman’s rank correlation). (b) Scatter plot for the propensity score-matched set (ρ = 0.01, P = 0.94; two-sided Spearman’s rank correlation). IVPCA: intravenous patient-controlled analgesia; NLR: neutrophil-to-lymphocyte ratio

In summary, both the complexity of POP mechanisms and the presence of multiple sources of postoperative inflammation in cardiac surgery likely account for the lack of association between preoperative inflammatory status and postoperative analgesic demand. Our scatter plot analysis showed no significant correlation between preoperative NLR and postoperative IVPCA consumption, even after adjusting for preoperative variables via propensity score matching (Figure 2). Although we used a preoperative NLR cutoff of 2.29, similar to thresholds reported in orthopedic [15] and orthognathic [7] surgery studies, this value does not appear applicable to cardiac surgery due to the distinct underlying pathophysiology. Therefore, preoperative NLR may not be a reliable predictor of postoperative analgesic consumption in cardiac surgery patients.

In recent decades, several studies have demonstrated the utility of NLR in predicting postoperative outcomes in cardiac surgery, including length of hospital stay [21], prolonged intubation, stroke, renal dysfunction [22], and mortality [23,24]. Among postoperative complications, immune dysregulation triggered by surgical trauma plays a critical role [21], and SIRS is a common mechanism underlying organ dysfunction in surgical patients [22]. Thus, preoperative NLR may serve as a more robust predictor of morbidity and mortality than of POP in cardiac surgery. However, in the present study, no association was found between preoperative NLR and clinical outcomes. Previous research has identified a preoperative NLR cutoff value of 3.0-3.5 as indicative of high mortality risk [24]; however, only 16 patients in our matched cohort had an NLR >3.5, limiting our ability to evaluate this association. A larger sample size with more patients in a high inflammatory state would be required to explore this relationship effectively.

Regarding comorbidities, patients with diabetes mellitus exhibited higher preoperative NLR values. Although a subset analysis was performed, preoperative NLR did not show a significant association with any IVPCA usage parameters. Diabetes mellitus is a chronic inflammatory condition, and elevated NLR has been linked to poor glycemic control, increased HbA1C levels, and higher mortality risk in diabetic patients [25]. Surgical patients with diabetes often require higher postoperative opioid doses, possibly due to increased pain sensitivity or decreased analgesic effectiveness [26]. While our findings did not show a significant association between preoperative NLR and postoperative NLR or outcomes in diabetic patients, future studies with larger cohorts are needed to clarify these relationships.

A key strength of this study is its focus on IVPCA consumption, an indirect yet objective indicator of POP. Our department has consistently used the same IVPCA regimen for more than two decades, ensuring uniformity in data collection. Despite this, IVPCA-based pain management remains uncommon in cardiac surgery compared to other surgical fields. Promoting patient autonomy through IVPCA in cardiac surgery may enhance postoperative recovery and outcomes.

However, the study has several limitations. First, pain scores were not analyzed, as they are not routinely recorded in our institution. Second, the IVPCA solution included multiple analgesics, making it difficult to assess the individual effects of each drug. We also did not evaluate the use of additional analgesics such as acetaminophen or NSAIDs. Third, since all patients underwent surgery with CPB, we could not isolate the influence of CPB on NLR levels. Therefore, it remains uncertain whether postoperative NLR elevation was due to surgical trauma alone or also influenced by CPB. Fourth, as a retrospective study, no standardized IVPCA protocol based on pain assessment was applied, and potential operator-dependent variability in IVPCA adjustments cannot be ruled out. Lastly, being a single-center study, the generalizability of our findings is limited.

## Conclusions

Preoperative NLR was not a predictor of postoperative IVPCA consumption or clinical outcomes in cardiac surgery. Therefore, it may not be a useful tool for determining IVPCA settings or estimating postoperative analgesic needs in this population. Future prospective studies are warranted to explore whether preoperative NLR could serve as a predictor when standardized analgesic regimens (e.g., NSAIDs and/or acetaminophen) and IVPCA settings are tailored based on pain scores in cardiac surgery patients.

## Appendices

Appendix A 

**Table 4:** Patient characteristics in diabetes mellitus Data are presented as mean ± SD, median (IQR), or counts (percentages). Continuous variables were compared using the Mann-Whitney U test or Student’s t test, while categorical variables were analyzed using the chi-squared test or Fisher’s exact test. Test statistics are reported as t, z, or chi-squared values, as appropriate. Alb: albumin; ASA-PS: American Society of Anesthesiologists Physical Status classification; BA: bronchial asthma; BSA: body surface area; Cre: creatinine; DM: diabetes mellitus; Hb: hemoglobin; HbA1c: hemoglobin A1c; Hct: hematocrit; HT: hypertension; LVEF: left ventricular ejection fraction; NLR: neutrophil-to-lymphocyte ratio

Variable	All patients with DM	Total set with DM				Matched set with DM			
		High preoperative NLR (<2.29)	Low preoperative NLR (<2.29)	Test statistics	P value	High preoperative NLR (2.29)	Low preoperative NLR	Test statistics	P value
Number	48	31	17			12	12		
Demographics									
Age (year)	73.1 ± 8.4	74.9 ± 8.2	71.6 ± 8.7	-1.3	0.2	75.5 ± 4.8	73.5 ± 9.5	-0.69	0.5
Male/female (n)	37/11	23/8	14/3	0.41	0.72	7/5	10/2	1.86	0.37
Height (cm)	161.1 ± 8.3	161.1 ± 8.9	161.1 ± 7.2	-0.03	0.98	156.0 ± 9.3	161.5 ± 7.3	1.61	0.12
Weight (kg)	60.7 ± 11.1	59.6 ± 10.8	62.8 ± 11.8	0.88	0.35	59.4 ± 11.6	62.1 ± 11	0.62	0.27
BMI (kg/m²)	23.3 ± 3.4	22.9 ± 3.5	24.1 ± 3.4	1.11	0.28	24.3 ± 3.8	23.7 ± 2.8	-0.43	0.67
BSA (m²)	1.64 ± 0.18	1.63 ± 0.18	1.67 ± 0.18	0.78	0.44	1.60 ± 0.19	1.67 ± 0.16	0.93	0.76
Comorbidities									
HT (n)	32 (66.7)	20 (64.5)	12 (70.6)	0.18	0.76	8 (66.7)	8 (66.7)	0	1
Smoker (n)	21 (44.7)	13 (43.3)	8 (47.1)	0.06	0.81	5 (41.7)	5 (41.7)	0	1
BA (n)	3 (6.3)	1 (3.2)	2 (11.8)	1.34	0.28	0 (0)	1 (8.3)	1	1
ASA-PS	4 (4-4)	4 (4-4)	4 (4-4)	0.53	0.6	4 (4-4)	4 (4-4)	0.6	0.76
LVEF (%)	64.5 (52.4-71.0)	65.0 (49.5-71.0)	64.0 (57.2-73.9)	-0.48	0.63	59.0 (34.0-67.0)	62.0 (48.8-77.6)	-0.06	0.98
Preoperative blood test									
WBC (×10³/μL)	5.9 (4.9-6.8)	5.8 (4.8-6.8)	6.0 (5.0-6.9)	1.27	0.86	5.8 (5.1 -6.1)	5.3 (4.9 -6.6)	0.29	0.8
Neutrophil (×10³/μL)	3.8 (2.9-4.6)	4.2 (3.3-4.8)	3.1 (2.6-3.8)	2.8	0.01	4.1 (3.4-4.6)	2.9 (2.3-3.4)	2.66	0.01
Lymphocyte (×10³/μL)	1.5 (1.0-1.9)	1.2 (0.9-1.5)	2.1 (1.6-2.5)	-4.62	<0.001	1.1 (0.9-1.5)	2.1 (1.5-2.5)	-3.44	<0.001
Hct (%)	37.3 (34.1-40.0)	37.1 (33.8-39.8)	38.8 (36.3-42.9)	-1.69	0.09	39.4 (37.1-40.2)	38.4 (36.2-43.5)	0.26	0.8
Alb (mg/dL)	4.0 (3.7-4.2)	3.9 (3.6-4.2)	4.1 (3.8-4.3)	-0.64	0.52	4.1 (3.7-4.5)	3.9 (3.7-4.1)	1.11	0.29
Cre (mg/dL)	1.1 (0.8-1.6)	1.3 (0.9-5.0)	0.9 (0.8-1.2)	2.15	0.03	0.9 (0.7-1.5)	1.0 (0.8-1.2)	-0.69	0.51
CRP (mg/dL)	0.18 (0.00-0.57)	0.18 (0.00-0.90)	0.23 (0.00-0.48)	0.4	0.69	0.12 (0.00-0.23)	0.06 (0.00-0.63)	-0.48	0.65
HbA1c (mg/dL)	6.4 (6.2-7.1)	6.4 (6.1-7.1)	6.6 (6.2-7.3)	-1.03	0.3	6.2 (6.0-6.7)	6.6 (6.2-7.3)	-1.62	0.12

Appendix B

**Table 5:** Anesthetic/surgical data and outcomes in patients with diabetes mellitus Data are presented as median (IQR) or counts (proportions). Continuous variables were compared using the Mann-Whitney U test, while categorical variables were analyzed using the chi-squared test or Fisher’s exact test, as appropriate. Statistical values are reported as t-values, z-values, or chi-square values. AVR: aortic valve replacement; CABG: coronary artery bypass graft; CPB: cardiopulmonary bypass; Delta NLR: each day of NLR minus preoperative NLR; DM: diabetes mellitus; IVPCA: intravenous patient-controlled analgesia; MVRR: mitral valve repair or replacement; NLR: neutrophil-to-lymphocyte ratio; POD: postoperative day; TAP: tricuspid annuloplasty

Variable	All patients with DM	Total set with DM				Matched set with DM			
		High preoperative NLR (<2.29)	Low preoperative NLR (<2.29)	Test statistics	P value	High preoperative NLR (>2.29)	Low preoperative NLR (<2.29)	Test statistics	P value
Number	48	31	17			12	12		
Anesthetic duration (min)	398 (346-477)	397 (352-457)	423 (335-505)	-0.29	0.77	371 (334-505)	424 (336-496)	-0.4	0.71
Surgical duration (min)	334 (281-402)	334 (281-391)	334 (268-425)	-0.28	0.78	296 (263-414)	345 (269-416)	0.78	0.43
CPB time (min)	150 (123-192)	151 (124-190)	148 (102-201)	0.41	0.68	161 (116-191)	154 (130-204)	0.06	0.95
Surgery type (n)				8.07	0.33			5.91	0.43
Valve									
AVR	11 (22.9)	9 (29.0)	2 (11.8)			5 (41.7)	2 (16.7)		
MVRR	2 (6.5)	2 (6.5)	0 (0.0)			1 (8.3)	0 (0.0)		
TAP	0 (0.0)	0 (0.0)	0 (0.0)			0 (0.0)	0 (0.0)		
Multi valve	8 (16.7)	6 (19.4)	2 (11.8)			1 (8.3)	2 (11.8)		
CABG	18 (37.5)	9 (29.0)	9 (52.9)			2 (16.7)	5 (41.7)		
CABG + valve									
CABG + AVR	6 (12.5)	3 (9.7)	3 (17.6)			2 (16.7)	2 (16.7)		
CABG + MVRR	1 (2.1)	0 (0.0)	1 (5.9)			0 (0.0)	1 (8.3)		
CABG + multi valve	1 (2.1)	1 (3.2)	0 (0.0)			1 (8.3)	0 (0.0)		
Others	1 (2.1)	1 (3.2)	1 (0.0)			0 (0.0)	0 (0.0)		
Intraoperative opioid administration									
Remifentanil (mcg/kg/min)	0.44 (0.34-0.51)	0.45 (0.34-0.51)	0.39 (0.34-0.45)	0.96	0.34	0.45 (0.34-0.51)	0.38 (0.34-0.44)	0.89	0.4
Fentanyl (mcg/kg/hr)	3.9 (2.9-6.0)	3.9 (2.7-6.1)	4.3 (3.1-56.0)	-0.29	0.77	3.9 (2.3-8.8)	4.6 (3.2-6.1)	0.06	0.95
Postoperative IVPCA usage									
Drug requirement in total (mL)	103.5 (83.7-161.5)	105.6 (84.9-162.6)	96.9 (79.2-164.0)	0.27	0.79	146.3.6 (88.1-188.0)	93.1 (70.9-119.0)	1.27	0.22
Usage duration (hours)	44.2 (38.6-64.3)	48.0 (41.8-64.9)	36.6 (38.1-44.2)	2.73	0.01	63.9 (40.0-62.4)	37.4 (38.2-58.5)	2.14	0.03
Number of bolus requests for the first 24 hours	2.0 (1.0-7.8)	2.0 (1.0-9.0)	3.0 (1.0-6.5)	-0.23	0.82	4.0 (0.3-11.8)	3.5 (2.0-5.0)	0.26	0.8
Number of bolus requests in total	9.0 (3.0-20.5)	11.0 (2.0-21.0)	8.0 (3.0-17.0)	0.27	0.79	12.0 (2.3-28.0)	8.5 (3.5-17.5)	0.32	0.76
Number of bolus delivery for the first 24 hours	2.0 (1.0-6.0)	2.0 (1.0-6.0)	3.0 (1.0-6.5)	-0.52	0.6	4.0 (0.3-9.5)	3.5 (2.0-5.0)	0.2	0.84
Number of bolus delivery in total	9.0 (3.0-17.8)	10.0 (2.0-18.0)	8.0 (3.0-15.0)	0.17	0.86	9.0 (2.3-25.5)	8.5 (3.5-15.8)	0.15	0.89
Outcomes									
Intubation duration (hour)	15.6 (13.1-18.9)	16.1 (14.3-19.4)	13.6 (9.3-17.5)	1.89	0.06	15.2 (13.2-19.2)	14.4 (12.9-22.9)	0.17	0.89
Length of ICU stay (day)	3.0 (3.0-4.0)	3.0 (3.0-4.0)	3.0 (2.0-3.0)	1.68	0.09	3.0 (2.3-3.8)	3.0 (2.3-3.0)	0.29	0.8
Length of hospital stay	16.0 (14.0-19.8)	16.0 (15.0-19.0)	14.0 (13.5-20.0)	0.92	0.36	15.5 (15.0-21.3)	14.5 (14.0-20.8)	0.55	0.58
Morbidity	9 (18.8)	6 (19.4)	3 (17.6)	0.02	1	2 (16.7)	3 (25.0)	0.24	1
Reoperation	0 (0.0)	0 (0.0)	0 (0.0)	1	1	0 (0.0)	0 (0.0)	-	-
Stroke	4 (8.3)	3 (9.7)	1 (5.9)	0.2	1	1 (8.3)	1 (8.3)	0	0
Renal failure	0 (0.0)	1 (3.2)	0 (0.0)	0.46	1	0 (0.0)	0 (0.0)	-	-
Prolonged intubation	8 (16.7)	5 (16.1)	3 (17.6)	0.02	1	2 (16.7)	3 (25.0)	0.24	1
Surgical site infection	0 (0.0)	0 (0.0)	0 (0.0)	-	-	0 (0.0)	0 (0.0)	-	-
Death	2 (4.2)	2 (6.5)	0 (0.0)	1.12	0.53	1 (8.3)	0 (0.0)	1	1
Delta NLR									
POD0	5.3 (2.8-13.6)	8.6 (4.3-21.8)	3.7 (2.4-12.4)	0.92	0.38	8.5 (-31.0-21.8)	3.7 (2.0-14.2)	0.19	1
POD1	11.5 (7.3-16.1)	11.0 (4.5-16.1)	11.7 (9.8-15.6)	-0.74	0.48	12.3 (16.2-25.3)	10.6 (9.9-17.5)	0.9	0.41
POD2	9.1 (6.0-14.1)	11.8 (5.5-25.3)	7.3 (6.1-10.1)	1.21	0.24	14.3 (10.0-32.8)	7.4 (6.5-10.6)	1.96	0.06
POD3	6.9 (5.0-10.9)	7.2 (5.3-13.0)	4.8 (3.0-8.4)	1.42	0.18	9.8 (6.8-26.0)	2.3 (1.3-4.2)	1	0.43
POD5	3.1 (1.7-5.1)	3.2 (1.7-6.6)	2.6 (1.3-4.0)	1.33	0.2	3.2 (2.8-6.1)	3.2 (1.5-5.7)	0.83	0.46
POD7	1.9 (1.2-5.2)	1.9 (1.1-5.2)	1.8 (1.2-5.2)	0	1	2.3 (1.1-5.2)	2.6 (1.5-4.5)	-0.65	0.57

Appendix C  

**Table 6:** Correlation between preoperative NLR and IVPCA usage/outcomes in patients with diabetes mellitus The relationship between preoperative NLR and postoperative parameters was analyzed using Spearman's rank correlation coefficient. Delta NLR: each day of NLR - preoperative NLR; IVPCA: intravenous patient-controlled analgesia; NLR: neutrophil-to-lymphocyte ratio; POD: postoperative day

Parameter	Total set with DM (n = 48)		Matched set (n = 24)	
	ρ	P value	ρ	P value
IVPCA usage				
Drug requirement in total	-0.02	0.91	0.24	0.26
Usage duration	0.33	0.02	0.41	0.05
Number of bolus requests for the first 24 hours	-0.23	0.12	-0.04	0.85
Number of bolus requests in total	-0.14	0.35	0.05	0.82
Number of bolus delivery for the first 24 hours	-0.24	0.1	-0.05	0.82
Number of bolus delivery in total	-0.15	0.3	0.01	0.96
Outcomes				
Intubation duration (hours)	0.25	0.09	0.11	0.6
Length of ICU stay (day)	0.2	0.18	-0.14	0.53
Length of hospital stay (day)	0.13	0.36	0.13	0.56
Delta NLR				
POD 0	0.36	0.11	0.26	0.45
POD 1	0.01	0.98	0.31	0.24
POD 2	0.17	0.38	0.32	0.23
POD 3	0.26	0.27	0.67	0.07
POD 5	0.3	0.13	0.02	0.96
POD 7	-0.07	0.74	-0.14	0.64
